# The relationship between effort-reward imbalance and quality of working life among medical caregivers: mediating effects of job burnout

**DOI:** 10.3389/fpsyg.2024.1375022

**Published:** 2024-07-25

**Authors:** Huang Qi, Sun Hongyan, He Song, Zhou Zhihang, Huang Ruiyin, Ma Youjia, Li Xia

**Affiliations:** ^1^Department of Gastroenterology, The Second Affiliated Hospital of Chongqing Medical University, Chongqing, China; ^2^School of Nursing, Southwest Medical University, Scichuan, China; ^3^Department of Emergency, Songshan General Hospital of Chongqing, Chongqing, China

**Keywords:** medical caregiver, effort-reward imbalance, job burnout, quality of working life, mediating effects

## Abstract

**Background:**

To determine the relationship between effort-reward imbalance (ERI) and quality of working life (QWL) among medical caregivers and the mediating role of job burnout.

**Methods:**

This was a cross-sectional survey. A total of 787 medical caregivers at seven hospitals from Sichuan and Chongqing, China, between May to September 2023 were included in this observational study. The General Information Questionnaire, Effort-Reward Imbalance Questionnaire (ERI), Maslach Burnout Inventory-General Survey (MBI-GS), and Quality of Working Life Scale (QWL7-32) were used for data collection. SPSS 26.0 and PROCESSv3.3 were used for all data analyses, including descriptive statistics.

**Results:**

A total of 820 questionnaires were distributed, of which only 787 were valid (return rate; 95.98%). The QWL score of medical caregivers was 126.94 ± 16.69. However, QWL scores were significantly different depending on age, number of children, family support status, department, years of experience, night shift status, number of night shifts per month, number of hours worked per day, monthly income, and occurrence of errors or adverse events (*p* < 0.05). Furthermore, job burnout and ERI were negatively correlated with QWL (*p* < 0.01). Job burnout mediated (95% CI = -0.365, −0.260) the relationship between ERI and QWL, accounting for 58.65% of the total effect.

**Conclusion:**

Medical caregivers have a medium level of QWL. Job burnout partially mediates the relationship between ERI and QWL. Medical caregiver managers can improve QWL by directly intervening in occupational stress and indirectly intervening in job burnout.

## Background

With the aging population and the surge in chronic illnesses, the demand for long-term care services, particularly formal and professional care during patient hospitalizations, has markedly risen (Ko et al., 2023). This trend has notably intensified the workload on medical caregivers, echoing the broader increase in work pressure experienced across various professions in the 21st century. In contemporary society, amidst a scarcity of nursing professionals and escalating expectations for high-quality care from patients, medical caregivers grapple with mounting work pressures. Within the nursing milieu, stressors such as demand overload, role conflict, shift work, and interpersonal challenges contribute to physical and mental exhaustion among nursing staff. Extensive evidence suggests that work stress is a significant predictor of fatigue (Huang et al., 2019; Jalilian et al., 2019; Liu et al., 2020), exacerbated by factors like prolonged exposure to patients, bodily fluids, and infectious diseases (Colindres et al., 2018). The severity of patients’ conditions, treatment protocols, and clinical complexities further amplify the burden on medical caregivers (Au et al., 2022; Yoo et al., 2023). Moreover, juggling multiple roles, medical caregivers often struggle to strike a balance between work and personal life (Kida et al., 2023).

The concept of effort-reward imbalance (ERI), introduced by Siegrist in the late 1990s, offers a classic framework for understanding job stress (Siegrist, 1996). It posits that when employees perceive a dissonance between their efforts expended and the rewards received, it can engender adverse outcomes such as job dissatisfaction, psychological distress, and physical health issues. Notably, this imbalance has been linked to feelings of anger, frustration, and perceived unfairness, triggering sustained activation of the autonomic nervous system and subsequently contributing to physical ailments (e.g., cardiovascular diseases) and mental disorders (Colindres et al., 2018). Prolonged exposure to work stress and reward imbalance emerges as pivotal factors in the physical and mental exhaustion experienced by medical caregivers (Sembajwe et al., 2012; Kowalczuk et al., 2020).

Job burnout, characterized by chronic stress manifesting as emotional exhaustion, depersonalization, and diminished personal accomplishment, represents a significant concern (Jacobson et al., 2022; Wright et al., 2022). Studies underscore its independent association with personal (e.g., gender, age) and work-related factors (e.g., working hours, administrative tasks) (Agarwal et al., 2020). Job burnout not only ensues from prolonged exposure to effort-reward imbalance but also profoundly shapes medical caregivers’ perceptions of their work environment and overall quality of life.

Quality of working life (QWL) encapsulates the perceived well-being of professionals serving others (Maslach et al., 1997; Li, 2003),and comprising elements such as job satisfaction, work-life balance, and overall mental health (Zhang et al., 2013a). Medical caregivers with enhanced QWL exhibit heightened job satisfaction, engagement, and resilience in navigating professional challenges, thereby fostering superior patient care experiences and outcomes (Bohm et al., 2021). Scholars posit that caregivers’ well-being is as integral as that of the patients they attend to Kalanlar and Kuru Alici (2020). The stress and strain of caregiving can impact caregivers’ physical and mental health, consequently influencing care quality and the recipients’ well-being (Siegrist, 1996; Li et al., 2001). The intricate relationship between healthcare workers’ QWL and effort-reward imbalance is influenced by myriad individual, organizational, and environmental factors.

Hence, this study aims to unravel the complex interplay between job burnout, quality of work life, and reward imbalance among healthcare workers. By scrutinizing the mediating role of job burnout in the nexus between effort-reward imbalance and well-being, this research endeavors to elucidate potential mechanisms for crafting organizational interventions and support structures aimed at ameliorating the adverse effects of job burnout and enhancing healthcare workers’ well-being in clinical settings. A comprehensive grasp of these dynamics empowers healthcare organizations to cultivate environments conducive to the flourishing of both healthcare providers and their patients.

## Methods

### Design and participants

This was a cross-sectional design study. A convenient sampling was used to select seven tertiary A hospitals from Sichuan Province and Chongqing in China based on the feasibility and accessibility of the survey. Data were collected between May and September 2023. The survey team was trained on how to understand and interpret the questionnaire after the survey plan and scales were determined to facilitate unified answers. The survey team consisted of the team leader, the initiator of the study, and three graduate students. The paper questionnaire was printed, sealed, and taken to the hospital where the participants were located. The medical caregivers were gathered in the conference hall of the hospital after obtaining the consent of the hospital administrator. The respondents were asked not to discuss the contents of the questionnaire during the study. The questionnaire was to be filled for about 10–30 min, and any questionnaire filled 10% of the time below or above this time was considered invalid. A total of 820 questionnaires were distributed, of which 787 were valid (return rate; 95.98%). Ten questionnaires did not meet the inclusion criteria, 17 were not completed, and 6 had regular answers (choose either all the first answers or all the last ones).

### Inclusion criteria

Only medical caregivers providing basic living care to patients employed directly by the hospital or indirectly through a third party were included. Moreover, only caregivers working 6 months or more yearly, who can read, comprehend and write were included in the study.

### Exclusion criteria

Medical caregivers who are hired by patient or patient’s family members; Those who exited in the middle of filling out the questionnaire.

### Measurement

#### Socio-demographic and work-related characteristics survey questionnaire

The socio-demographic and work-related characteristics questionnaire was developed by our team based on previous literature review and the descriptive interview of the medical caregivers. The questionnaire included socio-demographic factors (gender, age, education level, marital status, number of children, and family support) and work-related characteristics (department, years of working, position, night shifts, number of night shifts per month, daily-working hours, number of patients per shift and monthly income).

#### Effort-reward imbalance questionnaire, ERI

ERI questionnaire was first developed by Prof. Siegrist (Siegrist, 1996) and revised by Jian Li (Li et al., 2005). This questionnaire is widely used to assess occupation stress. In this study, two subscales of this questionnaire were used on an authorized basis. This questionnaire contained 17 items: ‘extrinsic effort’ (6 items), and ‘rewards’ (11 items). Furthermore, a 4-level scoring system (1 to 4 points representing “strongly disagree ~ strongly agree”) was used for the analysis. A reverse scoring was used for items 10, 11, 12 and 13. Scores on the “effort” scale (Cronbach α; 0.78) ranged from 6 to 24, with higher scores indicating more effort. The scores on the “reward” scale (Cronbach α; 0.81) ranged from 11 to 44 points, with higher scores indicating more rewards. Effort / reward ratio (ER Ratio) was used to reflects the state of ERI and was determined as follows: Effort/(Reward*0.5454). Ratio > 1, =1, and < 1 represented giving more than return, giving equal to return and giving less than return, respectively. The larger the ratio, the larger the imbalance and the more serious the occupational stress.

#### Maslach burnout inventory-general survey, MBI-GS

MBI-GS was first developed by Maslach et al. in 1996 (Maslach et al., 1997), and revised by Li chaoping and Shi kan, (Li et al., 2001; Li, 2003). MBI-GS is widely used to measure job burnout. This scale contains three subscales: emotional exhaustion (Ex, 5 items, Cronbach α; 0.88), cynicism (Cy, 5 items, Cronbach α: 0.83), and reduced personal accomplishment (Pe, 6 items, Cronbach α; 0.82). These items are scored on a 7-point frequency rating scale ranging from “0” (never) to “6” (daily). However, items 5, 7, 10, 11, 12, and 16 were evaluated using reverse scoring. Higher scores indicate more severe burnout.

#### Quality of working life scale, QWL7-32

QWL7-32 was developed by Zhang et al. (2013a, 2017). This scale is used to measure QWL (physical and psychological effect of a job on employees and employees’ feeling toward the job). The scale has seven dimensions, physical health (8 items, individual self-evaluation of their health), psychological health (5 items, anxiety, depression, tired of work, and other mental health), job satisfaction (8 items, satisfaction of remuneration, management and working environment), job pride (3 items, subjective feeling to professional values, professional brings the social status), perceived competence (2 items, feelings to whether work is handy), job initiative (4 items, level of positive and pleasure to work), sense of balance (2 items, evaluation of balance between career and family life). These items were scored on a 5-point frequency rating scale ranging from “1″ to “5″. However, items 3, 4, 7, 20, 22 ~ 27, and 29 were evaluated using reverse scoring. Higher scores indicate better QWL. The Cronbach α of QWL7-32 is 0.920 (Zhang et al., 2013b).

### Data analysis

**Figure 1:**
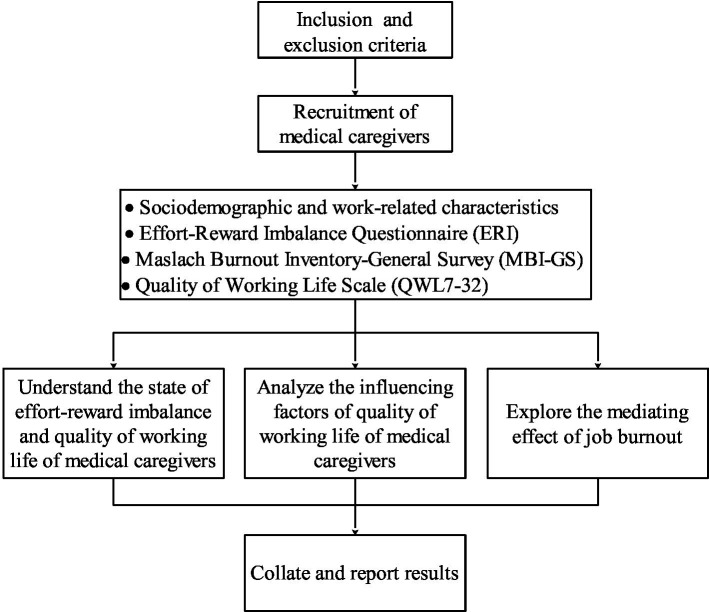
Flow of work.

Epidata3.1 and SPSS 26.0 were used for data entry management and statistical analysis, respectively. The normality of continuous variables (ERI scores, Job burnout scores, and QWL scores) were examined based on skewness and kurtosis tests. Descriptive analysis: Qualitative variables (nominal and ordinal), such as socio-demographic and work-related characteristics, were expressed as frequencies and percentages. Quantitative variables, such as the score of effort-reward imbalance, job burnout, and QWL, were expressed as mea*n* ± Standard deviation. One-way analysis of variance (ANOVA) was conducted using the t-test and ANOVA methods. For cases with homogeneous variance, ANOVA results were examined followed by post-hoc testing using the Turkey method. In cases where variance was not homogeneous and the sample size exceeded 50, the Welch results, specifically the Welch *F* value, were checked, and post-hoc testing was performed using the Games-Howell method Factors (*p* < 0.20) in single factor analysis were included in multiple linear regression analysis (stepwise method) tests to measure the effect of socio-demographic and work-related characteristics on QWL. The correlation among ERI, job burnout, and QWL was assessed using Pearson’s correlation analysis. PROCESS, developed by Hayes (2013), was installed in SPSS for the analysis of various types of mediation effects and moderation effects. The analyzed model (Model 4) was selected in template file (Hayes, 2013). Multiple linear regression analysis and PROCESSv3.3 were used to explore intermediary reconciliation paths. The Bootstrap method was used to repeatedly sample 5,000 times to detect the significance of the mediation effect and the moderated mediation effect. A two-sided test with *α* = 0.05 was used as the test level. *p* < 0.05 was determined as a statistically significant difference. The workflow is shown in Figure 1.

## Results

### Description of socio-demographic and work-related characteristics

A total of 787 medical caregivers were included in this survey. Most participants were females (*n* = 677, 86.02%), married (*n* = 708, 89.96%), and had a junior high school diploma (*n* = 497, 63.15%). About 92.38% (*n* = 727) of the participants were 40 years or older (Table 1). About 81.07% of the caregivers received family support (*n* = 638). The team system was the current management mode, including 643 team members (81.70%), 138 team leaders (17.53%), and 6 managers (0.76%). About 15.25% of medical nurses had worked for more than 10 years, of which 87.93% had undertaken night work (Table 1).

**Table 1:** QWL associations with medical caregivers’ sociodemographic and work-related characteristics *n* = 787 (100%).

Variables	*N* (%)	Mean (SD)	t/F	*P*
**Sociodemographic characteristics**				
Gender			1.650	0.099
Female	677 (86.02)	127.34 (16.50)		
Male	110 (13.98)	124.51 (17.67)		
Age (years)			4.037#	0.019*
≤39	60 (7.62)	128.33 (15.37)		
40 ~ 49	381 (48.41)	128.47 (16.05)		
≥50	346 (43.96)	125.02 (17.42)^b^		
Education level			1.269	0.282
Primary school and below	198 (25.16)	125.64 (17.01)		
Junior high school	497 (63.15)	127.66 (16.38)		
High school degree or above	92 (11.69)	125.84 (17.56)		
Marital status			2.554	0.054
Unmarried	8 (1.02)	111.50 (13.51)		
Married	708 (89.96)	127.12 (16.70)		
Divorced	52 (6.61)	125.88 (16.82)		
Death of a spouse	19 (2.41)	129.58 (14.55)		
Number of children			4.099#	0.013*
0	10 (1.27)	112.60 (13.44)		
1	289 (36.72)	127.74 (15.84)^a^		
2	437 (55.53)	126.99 (16.81)^a^		
≥3	51 (6.48)	124.84 (19.67)		
Sociodemographic characteristics				
Family support			13.703	<0.001*
Neutral	136 (17.28)	123.51 (16.76)		
Against	13 (1.65)	107.38 (19.13)^a^		
Support	638 (81.07)	128.07 (16.29)^a,b^		
work-related characteristics				
Departments			7.817#	<0.001*
Surgery department	440 (55.91)	127.42 (16.80)		
Emergency and critical care unit	51 (6.48)	130.63 (12.98)		
Gynaecology and obstetrics	29 (3.68)	135.41 (11.20)b		
Internal medicine	245 (31.13)	123.82 (17.40)^a,c,d^		
Others	22 (2.80)	132.45 (12.23)		
Years of working			3.207	0.041
<5^▲^	380 (48.28)	126.84 (16.02)		
5 ~ 10	287 (36.47)	125.69 (17.30)		
>10	120 (15.25)	130.26 (16.95)^b^		
Position			2.477	0.120
Members	643 (81.70)	127.26 (16.47)		
Leader	138 (17.53)	125.73 (17.89)		
Director	6 (0.76)	120.33 (7.74)		
**Work-related characteristics**				
Night shifts			2.696	0.007*
No	95 (12.07)	131.25 (15.86)		
Yes	692 (87.93)	126.35 (16.72)		
Number of night shifts per month (day)			20.588	<0.001*
0	95 (12.07)	131.25 (15.86)		
1 ~ 10	177 (22.49)	118.72 (15.90)^a^		
11 ~ 20	483 (61.37)	129.11 (15.93)^b^		
≥21	32 (4.07)	126.81 (20.03)^b^		
Daily working hours (h)			29.589	<0.001*
8 ~ 12 h	380 (48.28)	131.29 (16.30)		
12 h ~ 24 h	296 (37.61)	124.03 (15.47)^a^		
24 h	111 (14.10)	119.81 (17.11)^a,b^		
Number of patients per shift (cases)			1.520#	0.210
1 ~ 2	284 (36.69)	125.68 (18.03)		
3 ~ 4	339 (43.07)	128.34 (15.84)		
5 ~ 6	104 (13.21)	126.78 (15.25)		
>6	60 (7.62)	125.27 (16.87)		
Monthly income (yuan)			23.447#	<0.001*
≤3,000	106 (13.47)	116.01 (14.52)		
**Work-related characteristics**				
3,001 ~ 4,000	382 (48.54)	128.31 (16.83)^a^		
4,001 ~ 5,000	221 (28.08)	129.75 (14.53)^a^		
>5,000	78 (9.91)	127.15 (18.81)^a^		
Mistakes occurred			3.302	0.001*
No	424 (53.88)	128.75 (16.94)		
Yes	363 (46.12)	124.83 (16.16)		

Tukey’s honest significant difference test was used for the post-hoc test. *P<0.05; ^a^ Compare with group1, *P*<0.05; ^b^ Compare with group2, *P*<0.05; ^c^ Compare with group3, *P*<0.05; ^d^ Compare with group4, *P*<0.05; #Welch F.

^▲^Half a year ≤ years of working<5 years.

### Influencing factors of QWL

The differences in reported QWL outcomes based on six socio-demographic characteristics and nine work-related characteristics are shown in Table 1. Gender, education level, and marital status did not significantly impact QWL scores. However, three sociodemographic-related variables (age, number of children, and family support) significantly affected QWL scores. QWL scores were significantly lower among medical caregivers aged 50 or older (M = 125.02, SD = 17.42) than those aged 40–49 years old. Furthermore, the QWL scores were higher among participants receiving family support than those without (M = 128.07, SD = 16.29). QWL scores were lowest among participants without children (M = 112.60, SD = 13.44). Position and number of patients per shift did not significantly affect QWL scores. However, seven work-related characteristics (departments, years of working, night shifts, number of night shifts per month, daily working hours, monthly income, and mistake occurrence) significantly affected QWL scores. For example, medical caregivers with >10 years of work experience had significantly higher QWL scores than those with 5–10 years of work experience (M = 130.26, SD = 16.95). QWL scores were significantly lower among participants with night shifts than those without (M = 131.25, SD = 15.86).

### ERI and QWL scores

The QWL of the caregivers was at a moderate level (QWL score; 126.94) (Table 2). The average ERI score was 0.91. In addition, 222 patients had ERI (detection rate; 28.21%), also known as professional stress.

**Table 2:** Medical caregivers’ level of effort-reward imbalance and QWL n =787.

Variables	Mean (SD)
Effort-reward imbalance^△^	0.91 (0.24)
QWL	126.94 (16.69)
Physiology	33.28 (4.32)
Psychological	18.61 (4.60)
Satisfaction	32.31 (5.28)
Pride	10.98 (2.67)
Sense of Competence	8.03 (1.80)
Proactivity	16.67 (3.22)
Sense of balance	7.05 (1.77)

^△^E/R (ERratio).

### Multiple linear regression analysis

Variance Inflation Factor (VIF) close to 10 indicates severe collinearity among variables (Pallant, 2020). In this study, VIFs of all variables were smaller, indicating less collinearity. Multiple linear regression analysis was conducted with the quality of occupational life as the dependent variable and 12 variables (departments, gender, age, marital status, number of children, family support, working years, night shifts, number of night shifts per month, working hours per day, monthly income, and appearance) as independent variables. The regression equation showed statistical significance (*F* = 22.964, *P*<0.001, adjusted $R^{2}\text{;}$ 0.218). Number of night shifts per month, mistake occurrence, departments, family support, daily working hours, and monthly income affected the quality of life of medical nurses. Compared with participants with families with a neutral attitude and a monthly income of ≤3,000 yuan, participants with a supportive family and higher monthly income had higher QWL scores. Moreover, participants with no night shift, no errors or accident occurrence, with a family with a neutral attitude, and those receiving obstetrics and gynecology had higher QWL scores. Compared with caregivers working 8–12 h daily, participants with night shifts of 1–10 days per month, experiencing mistakes or accidents, working in internal medicine, do not have family support and have longer working hours had lower QWL (Table 3).

**Table 3:** Multiple covariance regression analysis of factors influencing the quality of working life of medical caregivers.

	B	*SE*	Beta	*t*	*P*	*VIF*
(constant)	123.88	2.045	–	60.573	<0.001	-
Family support	4.124	1.406	0.097	2.933	0.003	1.097
Monthly income (3,001 ~ 400yuan)	10.366	1.661	0.311	6.241	<0.001	2.493
Monthly income (4,001 ~ 5000yuan)	9.974	1.837	0.269	5.431	<0.001	2.463
Monthly income (>5000yuan)	9.634	2.294	0.173	4.199	<0.001	1.700
Number of night shifts per month (1 ~ 10 day)	−8.859	1.314	−0.222	−6.741	<0.001	1.089
Mistakes occurred	−5.436	1.087	−0.163	−4.999	<0.001	1.063
Departments (Internal medicine)	−2.346	1.160	−0.065	−2.023	0.043	1.043
Family against	−16.371	4.343	−0.125	−3.769	<0.001	1.108
Daily working hours (12 ~ 24 h)	−6.242	1.154	−0.181	−5.407	<0.001	1.131
Daily working hours (24 h)	−8.651	1.677	−0.181	−5.158	<0.001	1.232

$R^{2}$ = 0.228; $\Delta R^{2}$ = 0.218; F = 22.964, *P*<0.001;”-” blank item; reference information: family does not support or object, monthly income ≤ 3,000 yuan, 0 days of night shift per month, no mistakes or bad accidents, obstetrics and gynecology, working hours 8–12 h a day.

### Bivariate correlation between the QWL and ERI, and between the QWL and job burnout

The bivariate correlations between ERI and QWL and between Job burnout and QWL were analyzed using Pearson correlation coefficients (Table 4). Pearson correlation showed that ERI and job burnout were negatively correlated with QWL (*p* < 0.001).

**Table 4:** Bivariate correlation between the QWL and effort-reward imbalance, and the QWL and the job burnout of medical caregivers.

	QWL
Effort-reward imbalance	*r* = −0.533**, *P*<0.001
Job burnout	*r* = −0.673**, *P*<0.001

**Correlation is significant at the 0.01 level (2- tailed).

### The mediating effect of job burnout on the relationship between ERI and QWL

**Figure 2:**
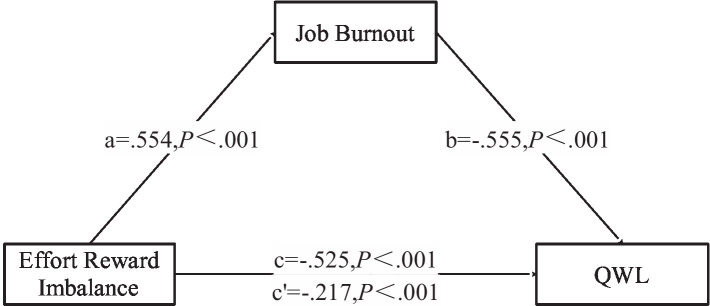
Mediating effect of job burnout on the relationship between effort-reward imbalance and QWL.

The mediating effect of job burnout on the relationship between ERI and QWL was evaluated using mediation model analysis. Gender, age, educational background, years of working, and monthly income can affect the mediating effect of job burnout between ERI and QWL and thus were included in the model as control variables. The results showed that ERI significantly impacted QWL ( β = −0.525, 95% CI = -0.586, −0.463, SE = 0.031, t = − 16.795, *P*<0.001) and job burnout ( β =0.554, 95% CI = 0.496, 0.613, SE = 0.030, t =18.530, *P*<0.001). Moreover, ERI and job burnout significantly affected QWL (*P*<0.001), indicating that job burnout mediates the relationship between ERI and QWL (Table 5). Further bootstrap results showed that job burnout partially mediated between the ERI and QWL (58.65%; 95% CI = −0.356, −0.260) (Tables 5, 6 and Figure 2).

**Table 5:** The mediating effect of job burnout between effort-reward imbalance and QWL.

Outcome variable	Control variable	*R^2^*	*F*	*β*	*t*
QWL		0.295	54.256(6)***		
	Gender			−0.043	−0.483
	Age			0.010	0.196
	Education background			0.106	2.024*
	Years of working			0.045	1.050
	Monthly income			0.084	2.282*
	**Effort-reward imbalance**			−0.525	−16.795***
Job burnout		0.353	70.782(6)***		
	Gender			0.050	0.588
	Age			0.127	2.581*
	Education background			0.076	1.517
	Years of working			0.011	0.278
	Monthly income			−0.080	−2.253*
	**Effort-reward imbalance**			0.554	18.530***
QWL		0.494	108.578(7)***		
	Gender			−0.015	−0.201
	Age			0.081	1.843
	Education background			0.148	3.335**
	Years of working			0.051	1.413
	Monthly income			0.040	1.276
	**Effort-reward imbalance**			−0.217	−6.827***
	**Job burnout**			−0.555	−17.517***

*P<0.05; **P<0.01; ***P<0.001.All data in this model are standardized data. (6) There are 6 control variables; (7) There are 7 control variable; $R^{2}$ :R-square; β : standardized regression coefficients.

**Table 6:** Mediation effect decomposition table.

	*β*	BootSE	BootCI	Ratio (%)
Direct effect	−0.217	0.034	−0.285, −0.151	41.35%
Indirect effect	−0.308	0.025	−0.356, −0.260	58.65%
Total effect	−0.525	0.033	−0.283, −0.153	

## Discussion

Medical caregivers can meet the basic living care needs of inpatients during hospitalization. Therefore, the psychological status and QWL of caregivers are crucial for the healthy development of medical caregivers and the provision of high-quality services to patients (Li et al., 2001; Kalanlar and Kuru Alici, 2020). In this study, results showed that the QWL score of medical caregivers was at the intermediate level since the QWL scores were closer to the median QWL. Further analysis revealed that socio-demographic and work-related characteristics affect QWL. e.g. QWL scores were significantly lower among participants with night shifts, but QWL scores were higher among participants receiving family support. Similarly, another research (Thakre et al., 2017; Alharbi et al., 2019; Baysal and Yildiz, 2019; Salahat and Al-Hamdan, 2022) showed that the QWL of employees is mainly at the middle level. The QWL of doctors in Chinese public hospitals is higher than that of medical caregivers (Tao et al., 2022). The decisions and judgments made by doctors are very critical during patient treatment. Treatment options are directly related to patient disease progression, treatment outcomes, and patient safety. As a result, doctors bear relatively greater pressure and risk factors than caregivers. Particularly, medical costs are high, and patients have higher expectations, indicating that the doctor-patient relationship may be damaged and threatened if the treatment results do not meet patients’ expectations, thereby affecting the doctor’s QWL (Tao et al., 2022).

### The impact of socio-demographic characteristics on the QWL

Univariate analysis showed that age, number of children, and family support could impact QWL (*p* < 0.05). In addition, regression analysis showed that only family support significantly affected QWL, indicating that medical caregivers who are supported by family members have higher QWL. Similarly, Thakre et al. (2017) showed that QWL of nurses is positively related to family support. Parra-Giordano D (Parra-Giordano et al., 2022) interviewed seven assistant nurses via qualitative interviews and found that good work resources, work environment, close family relationships improve QWL. Furthermore, family support is positively related to QWL in various populations, including chronically ill patients (Jiang et al., 2022; Mphasha et al., 2022), students (Xian et al., 2022; Yang et al., 2022), and teachers (Di et al., 2023). Family care and social support can enhance employees’ confidence and sense of competence at work, making it easier to overcome difficulties. Family members can understand and accept the working model of medical caregivers, and make up for or forgive their lack of family care, thus improving their output. Family Supportive Supervisor Behavior (FSSB) is defined as behaviors exhibited by supervisors that support employees’ families: emotional support, instrumental support, role modeling behaviors, and creative work-family management (managerial-initiated actions to restructure work to facilitate employee effectiveness on and off the job) (Hammer et al., 2009, 2013). FSSB is an important link among employee, family support, and work (Hammer et al., 2009). FSSB is negatively correlated with work–family conflict, employee turnover and positively correlated with job satisfaction (Hammer et al., 2009). A randomized controlled study (Hammer et al., 2011) showed that the physical health and job satisfaction of employees with high work–family conflicts improve when supervised by managers who receive FSSB training. Hence, it is recommended that managers undergo FSSB training to enable them to better support their employees in managing their family roles. This, in turn, can contribute to enhancing the QWL among medical caregivers.

### Analysis of the effect of work-related characteristics on QWL

In this research, results showed that seven work-related factors (departments, years of working, night shifts, number of night shifts per month, daily working hours, monthly income, and mistakes) could impact QWL (*p* < 0.05). In addition, regression analysis found that monthly income, number of night shifts per month, mistake occurrence, department, and daily working hours significantly affect QWL. The higher the monthly income, the higher the QWL score of medical caregivers, and the better the QWL. Compensation also affects QWL of employees, consistent with Almugren and Zedan (2022) study. Monthly income and welfare benefits are positively associated with QWL. The higher the income, the more capital medical caregivers can invest in health and improve family life, thus improving QWL. Remuneration is the recognition of work performance. Higher remuneration can motivate employees and enhance their confidence and job satisfaction. Therefore, managers or government departments should improve QWL of medical caregivers by improving their salaries or implementing a reasonable welfare system. Furthermore, QWL score decreased with an increasing number of night shifts per month and daily working hours. Similarly, Asaoka et al. (2013) reported night work, long night work hours, and delayed circadian rhythms cause sleep disturbances among nurses. Night shifts are a long-term unavoidable adverse stimulus, which is associated with unhealthy lifestyles among caregivers based on the physiological dimension of occupational quality of life (Shan et al., 2018). The QWL of medical caregivers decreases with the increasing number of night shifts. Furthermore, QWL is decreased among medical nurses with night shifts of 1–10 days per month due to imbalanced health investment and income returns. Long daily working hours and a 24-h working mode can cause stress at work, leading to double physical and psychological pressure among medical caregivers. Long working hours affect work-family balance, thus influencing QWL. Therefore, managers should schedule reasonable shifts and allocate reasonable work to improve physical and mental health of medical caregivers, thus enhancing job satisfaction and initiative and QWL. Medical caregivers with errors in previous jobs also have lower QWL scores. Therefore, professional and complete training should be conducted to minimize mistakes. Second, a sound emergency system should be established for prompt and active response when errors occur. Furthermore, a sound and scientific reward and punishment system should be established to regulate the behavior of employees. Finally, the risk faced by the patient should be minimized after a mistake has occurred. Moreover, managers should assess the cause based on experience summarization to prevent the occurrence of similar mistakes.

### The mediating role of job burnout between ERI and QWL

In this study, socio-demographic characteristics did not affect QWL. In addition, ERI directly affected the impact of work-related characteristics on QWL, while Job burnout indirectly affected the impact of work-related characteristics on QWL. Job burnout had a partial mediation effect between ERI and QWL (*β* = −0.308; 95% CI: −0.356, −0.260), accounting for 58.65%. The mediating effect of job burnout has also been confirmed among teachers (Wang et al., 2020), doctors (Feng et al., 2022), nurses (Song et al., 2021), new generation of employees (Yu et al., 2022), and bus drivers (Tu et al., 2021). Job burnout is a feeling stimulated by long-term occupational pressure, characterized by physical and mental exhaustion, emotional exhaustion, apathy, alienation from others, decreased work initiative and enthusiasm, and a decreased sense of personal accomplishment (Edú-Valsania et al., 2022). The Job Demand-Resource Model (JD-R) divides work conditions into two broad categories (Demerouti et al., 2001; Lesener et al., 2020): job needs and job resources. Work demand refers to the social or organizational purpose of work that should be achieved through physical or mental efforts, indicating that demand overload may lead to physical or psychological fatigue. Work resources, also known as health protection factors, include physical, psychological, and social factors. Exhaustion and cynicism are the major symptoms of burnout symptoms (Huang and Simha, 2018). Exhaustion is a feeling of overstretched and depleted resources, while cynicism is a negative or callous response to job responsibilities (Gan et al., 2019). Specifically, the time and energy spent by medical caregivers participating in training and caring for patients and their career development prospects, advancement opportunities, respect, and job security is imbalanced, further increasing the risk of burnout. A study of burnout and suicidal behavior among health professionals in Portugal noted that burnout had a positive effect on the frequency of suicidal behavior, especially among health care workers. Active prevention of non-occurring burnout and active intervention of ongoing burnout are particularly important for the occupational health of health care workers (Jesus et al., 2023). Consequently, it is crucial for managers to mitigate job burnout as a means to enhance work enthusiasm and initiative, ultimately leading to an improvement in the quality of work-life (QWL).

## Conclusion

Healthcare workers have a moderate QWL. Moreover, the QWL of healthcare workers is influenced by demographic variables and job-related factors. ERI can increase job burnout and decrease QWL. Also, job burnout mediates the relationship between ERI and QWL. Therefore, managers should reduce ERI to reduce job burnout and improve QWL.

## Limitations

The study was conducted in a specific region of China, which creates geographical limitations. Study participants were recruited from general hospitals, and health care workers in specialty hospitals were not included in the survey. We look forward to addressing these gaps in future surveys. In addition, the study used a cross-sectional design that excluded the evaluation of temporal and causal relationships between variables. All results were assessed using a self-reported questionnaire, which may introduce recall bias. We will continue to improve in the follow-up study.

## Data availability statement

The raw data supporting the conclusions of this article will be made available by the authors, without undue reservation.

## Ethics statement

The studies involving humans were approved by the Institutional Review Board Committee of Ethics Committee of the Second Affiliated Hospital of Chongqing Medical University. The studies were conducted in accordance with the local legislation and institutional requirements. The participants provided their written informed consent to participate in this study.

## Author contributions

HQ: Writing – review & editing, Writing – original draft, Software. SH: Writing – review & editing, Project administration, Investigation. HS: Writing – review & editing, Methodology, Formal analysis. ZZ: Writing – review & editing, Supervision, Software. HR: Writing – review & editing, Investigation. MY: Writing – review & editing, Methodology, Investigation. LX: Writing – review & editing, Writing – original draft, Supervision, Project administration, Data curation.
